# Web-Based Independent Versus Laboratory-Based Stop-Signal Task Performance: Within-Subjects Counterbalanced Comparison Study

**DOI:** 10.2196/32922

**Published:** 2022-05-30

**Authors:** Antoinette Poulton, Li Peng Evelyn Chen, Gezelle Dali, Michael Fox, Robert Hester

**Affiliations:** 1 Melbourne School of Psychological Sciences University of Melbourne Parkville Australia

**Keywords:** Stop-Signal Task, response inhibition, inhibitory control, online assessment, web-based assessment, cognition

## Abstract

**Background:**

Considered a facet of behavioral impulsivity, response inhibition facilitates adaptive and goal-directed behavior. It is often assessed using the Stop-Signal Task (SST), which is presented on stand-alone computers under controlled laboratory conditions. Sample size may consequently be a function of cost or time and sample diversity constrained to those willing or able to attend the laboratory. Statistical power and generalizability of results might, in turn, be impacted. Such limitations may potentially be overcome via the implementation of web-based testing.

**Objective:**

The aim of this study was to investigate if there were differences between variables derived from a web-based SST when it was undertaken independently—that is, outside the laboratory, on any computer, and in the absence of researchers—versus when it was performed under laboratory conditions.

**Methods:**

We programmed a web-based SST in HTML and JavaScript and employed a counterbalanced design. A total of 166 individuals (mean age 19.72, SD 1.85, range 18-36 years; 146/166, 88% female) were recruited. Of them, 79 undertook the independent task prior to visiting the laboratory and 78 completed the independent task following their laboratory visit. The average time between SST testing was 3.72 (SD 2.86) days. Dependent samples and Bayesian paired samples *t* tests were used to examine differences between laboratory-based and independent SST variables. Correlational analyses were conducted on stop-signal reaction times (SSRT).

**Results:**

After exclusions, 123 participants (mean age 19.73, SD 1.97 years) completed the SST both in the laboratory and independently. While participants were less accurate on go trials and exhibited reduced inhibitory control when undertaking the independent—compared to the laboratory-based—SST, there was a positive association between the SSRT of each condition (*r*=.48; *P*<.001; 95% CI 0.33-0.61).

**Conclusions:**

Findings suggest a web-based SST, which participants undertake on any computer, at any location, and in the absence of the researcher, is a suitable measure of response inhibition.

## Introduction

Considered a facet of behavioral impulsivity, response inhibition refers to the capacity to withhold, interrupt, or delay a prepotent behavioral response and is a key element of executive function [1-4]. Also termed “inhibitory control” or cognitive control,” it facilitates adaptive and goal-directed behavior [5]. The Stop-Signal Task (SST), a commonly employed measure of response inhibition [6], has been used to examine the inhibitory control of healthy adults and children [7-9], older adults [10], and clinical groups [11]. It is routinely used to show how individuals diagnosed with attention-deficit/hyperactivity disorder (ADHD) [12] and substance use disorders (SUDs) [13,14] tend to be characterized by heightened impulsivity. While there is abundant literature examining the psychometrics of the task when it is undertaken on dedicated computers under laboratory conditions [15-17], it is unclear if performance on web-based versions of the task differs as a function of the testing environment.

Although there are several variants of the SST, they all fundamentally assess the ability to suppress a motor response that has already been initiated [18-21]. In all cases, individuals must respond rapidly to frequently appearing (go) stimuli but inhibit responses to others (stop signals) presented much less often [17,21-23]. The imbalance in the occurrence of each type of stimulus creates a response prepotency that manifests in a difficulty inhibiting responses when required. The go component of the SST is essentially a 2-choice reaction time (RT) task that involves the electronic presentation of 1 of 2 stimuli (for example, X or O). In response, participants are required to press the corresponding letter on a keyboard as quickly as possible—this generates a go RT. The stop component of the task typically occurs on 25% of trials and comprises the presentation of a stop signal—in the form of an auditory tone or visual indicator—designed to inform participants that they must withhold (or inhibit) their response to the stimulus on that trial. The period between the presentation of the go stimulus and the stop signal is known as the stop-signal delay (SSD) [21,24]. Although initially usually set at 250 milliseconds, the onset of the SSD varies dynamically in a stepwise manner on each trial and as a function of participant performance. In this way, successful inhibition approaches 50% accuracy by the end of the task. Stop-signal reaction time (SSRT) is often the main variable of interest in the SST and represents the difference between mean go RT and the average SSD [20,21].

The SST is typically programmed using common software packages and has traditionally been presented on stand-alone computers in controlled research settings [23]. This assists in ensuring that task presentation is consistent across participants and variability—related both to computer hardware or software and the testing environment—is minimal. Participants are thus generally required to visit the laboratory in order to take part in studies using this tool. They may even be tested individually. This gives rise to two potential limitations: sample size becomes a function of cost or time constraints, and sample diversity is restricted to those willing and able to attend the laboratory. In turn, this may impact power and means findings may not be generalizable to the wider population. Moreover, COVID-19 restrictions have meant that in-person testing is frequently unavailable or hampered by the need to implement social distancing, cleaning or sanitizing, and personal protective equipment protocols. While such procedures may have unintended consequences that impact the quality of the data, they are also likely to be costly and time-consuming [25]. This may further exacerbate sample size and diversity issues.

These limitations may potentially be overcome through web-based testing. While there has been a substantial increase in the popularity of using the internet as a medium for conducting research in social psychology—which appears to have resulted in larger samples and increased statistical power [26]—this trend has been less evident in the cognitive arena, possibly owing to validity and reliability concerns [27,28]. Nonetheless, researchers have, more recently, begun to examine whether participants perform in similar ways when undertaking web-based cognitive tasks independently versus in the laboratory [29-32]. Results of these studies suggest that while main effects remain the same, there might be some timing and accuracy offsets related to participant concentration and hardware or software variability in uncontrolled testing environments [29,31]. To date, the SST has not been the subject of such an investigation. The aim of this study was therefore to investigate whether performance on a web-based version of the SST differed as a function of the testing environment. Data were collected prior to the onset of the COVID-19 pandemic. Informed by findings in similar previous studies [29,31], we hypothesized the independent web-based SST—that is, the SST performed outside the laboratory, on any computer, and in the absence of any researcher—would be characterized by decreased go and stop accuracy, increased go omissions and go errors, and longer SSRTs, as compared to the laboratory-based SST carried out on the web. Additionally, relative to the laboratory-based SST, intraindividual variability would be greater in the independent task. Nonetheless, given that other studies have also found acceptable comparability between independent and laboratory-based cognitive tasks [29-32], we expected that there would be a robust positive relationship between independent and laboratory-based SSRTs.

## Methods

### Recruitment and Procedure

Participants were 166 individuals (mean age 19.72, SD 1.85, range 18-36 years; 146/166, 88% female) who completed this study as part of their undergraduate psychology studies. First-year psychology students at the University of Melbourne are encouraged to take part in studies being conducted within the School of Psychological Sciences. Students receive course credit as reimbursement for their time.

On signing up for the study (via a School of Psychological Sciences research participation landing page), participants were randomly assigned to either first complete the web-based SST in the laboratory or independently. The independent condition was thus completed in counterbalanced order, with half of the participants undertaking the task prior to visiting the laboratory and the other half doing it following their laboratory visit. In both cases, consent was obtained via a web-based form, and links to the task were emailed to the participants. In the laboratory, participants also completed alcohol and substance use surveys.

### Measures

#### Substance Use

Participants completed the Alcohol Use Disorders Identification Test (AUDIT), which assesses alcohol intake, problems, and dependence with reference to the preceding 6 months [33]. Harmful use of licit and illicit drugs was assessed using the Alcohol, Smoking and Substance Involvement Screen Test (ASSIST), which assesses frequency of use and associated problems over the previous 3 months [34].

#### Inhibitory Control

**Figure 1:**
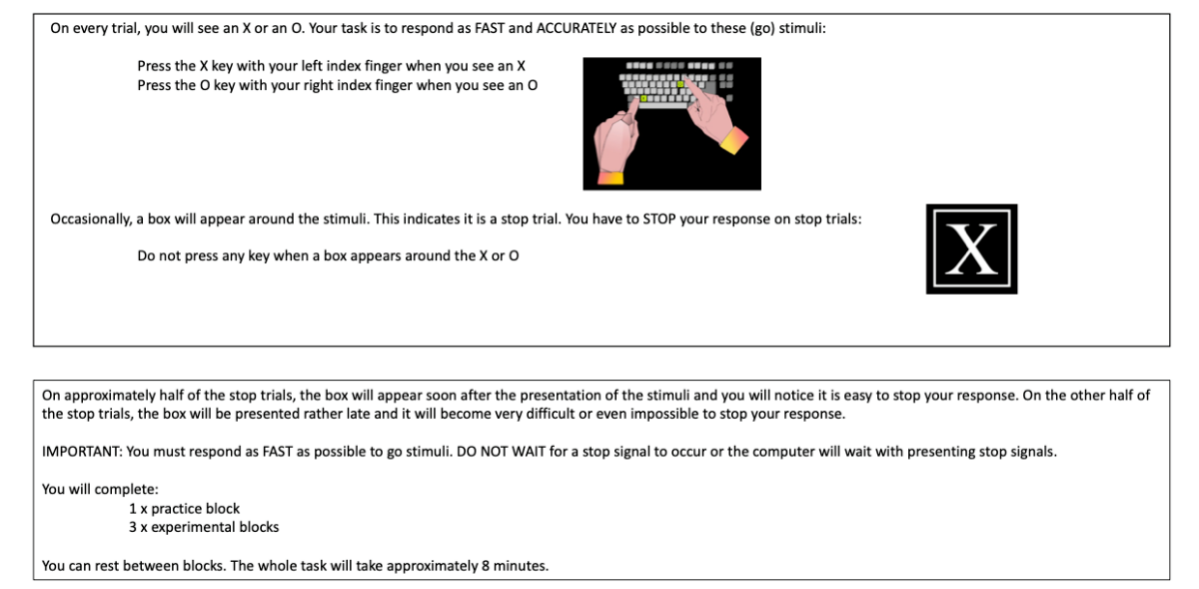
Initial instructions (provided across 2 screens) for the web-based Stop-Signal Task.

**Figure 2:**
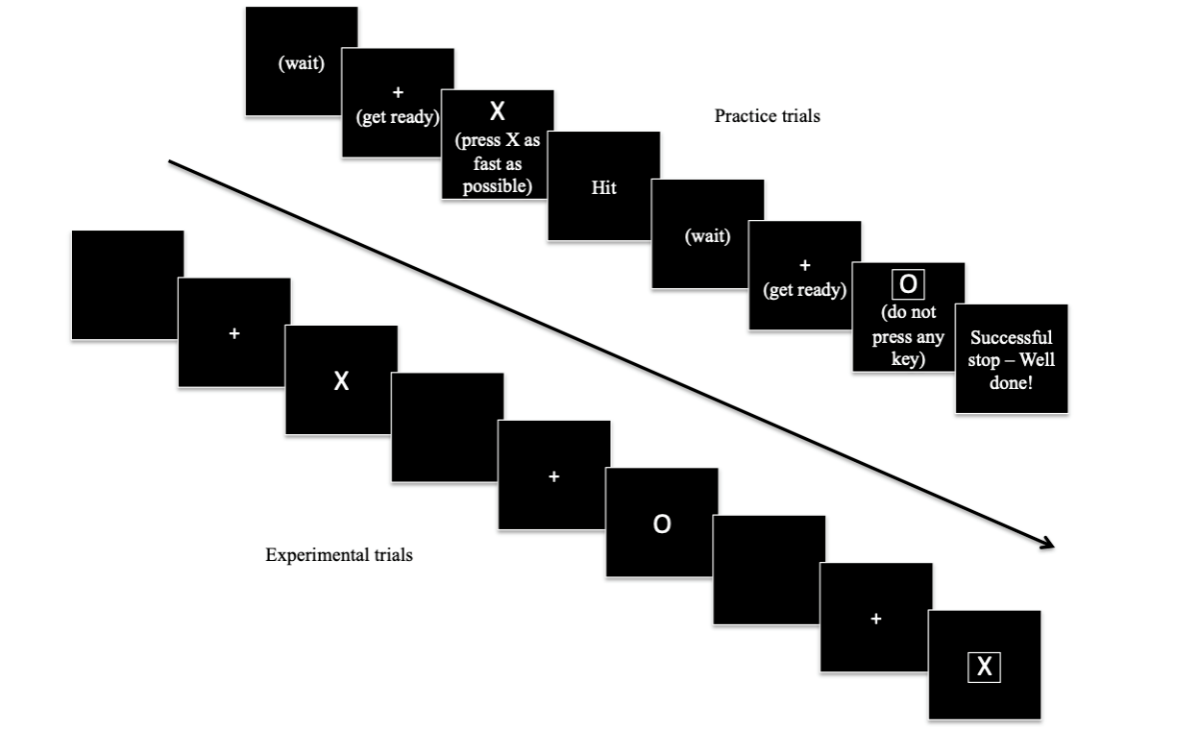
Schematic representation of Go and Stop trials in the practice and experimental blocks of the web-based Stop-Signal Task. Practice trials have an inter-trial interval (ITI) of 4250 milliseconds and comprise a blank screen (1000 milliseconds), fixation cross (250 milliseconds), stimulus presentation (1000 milliseconds), and feedback screen (2000 milliseconds). Experimental trials have an ITI of 2250 milliseconds and comprise a blank screen (1000 milliseconds), fixation cross (250 milliseconds), and stimulus presentation (1000 milliseconds).

The web-based SST was programmed using HTML (version 5) and JavaScript client-side along with PHP and MySQL server-side for data storage and management [35]. The task is run on Windows or Mac desktop or laptop computers and supported by all major browsers. Initial instructions are provided across 2 screens in a white 20-point Sans Serif font on a black background (Figure 1). The task consists of a practice block of 32 trials and 3 blocks of 64 experimental trials. Practice trials have an intertrial interval (ITI) of 4250 milliseconds, while experimental trials have ITI of 2250 milliseconds (Figure 2). The fixation cross and stimuli are rendered in 100- and 150-point Serif font, respectively. Trial-by-trial feedback is provided during the practice block, while block-based feedback is given during experimental trials. Within-task prompts or feedback are provided in white and colored 25-point Serif font on a black background. During the practice block, the following trial-based prompts or feedback are provided:

- Fixation cross screen: *Get ready*
- Go stimuli presentation screen: *Press X (or O) as fast as possible*
- Stop stimuli presentation screen: *Do not press any key*
- Successful go with response time < 500 milliseconds: *Hit* (green text)
- Successful go with response time ≥ 500 milliseconds: *Hit (but try to go faster)* (yellow text)
- Go omission: Miss *(you must go faster)* (red text)
- Incorrect go: Miss *(incorrect keystroke)* (red text)
- Following three consecutive go omissions: *WARNING: You MUST respond to X/O go stimuli as fast as possible* (red text)
- Successful inhibition on a stop trial: *Successful stop – Well done!* (green text)
- Unsuccessful inhibition on a stop trial: *Unsuccessful stop – try not to respond to stop trials!* (red text)
- Blank screen: *Wait*

During experimental blocks, the only trial-based prompt provided occurs if participants neglect to respond to 3 consecutive go trials. In this case, participants are warned: *You MUST respond to X/O go stimuli as fast as possible* (red text). At the end of both the practice and experimental blocks, participants are provided with the following block-based feedback: number of incorrect responses to go stimuli; number of missed responses to go stimuli; mean reaction time to go stimuli (where this is ≥500 milliseconds, participants are warned *Too slow! Respond faster*); percentage of correctly suppressed responses on stop trials; and seconds left to wait (10-second countdown to the next block).

Go stimuli comprise random presentation of letters X or O that map to corresponding keyboard letters. A stop signal in the form of a white box surrounding the go stimuli appears on 25% of randomly selected trials. Stop signals are not presented on consecutive trials. The initial SSD is set at 250 milliseconds and adjusts dynamically as a function of the participant response; successful inhibitions result in a 50-millisecond increase in the SSD, while unsuccessful inhibitions decrease it by 50 milliseconds. Variables of interest may include go accuracy, omissions, and errors; average go RT; intraindividual SD; stop accuracy; mean SSD; average RT on unsuccessful stop trials; and SSRT [21]. SSRT is derived when the mean SSD is subtracted from average go RT; greater SSRTs denote reduced inhibition ability [22]. Participants are excluded if the mean RT of either correct or incorrect failed stops (ie, failed stops where the key press does or does not respectively accord with the stimulus) is greater than the mean go RT [21]. They are also excluded if the stop accuracy is less than 25% or greater than 75%, go errors are greater than 10%, or if SSRT is less than 50 milliseconds [17].

### Data Analysis

**Figure 3:**
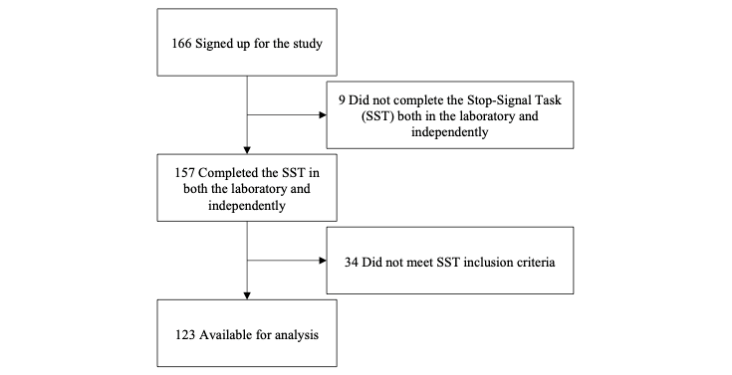
Study Participation Flowchart.

To achieve a medium to small effect with α=.01, an a priori power analysis conducted using G*Power 3.1 [36] indicated a total sample size of 115 was required to achieve a power of 80%. A greater number of participants (ie, n=166) were recruited to account for potential exclusions. Participants who did not complete both components of the study (n=9) were excluded from the analysis; this left 79 participants who had undertaken the independent task prior to visiting the laboratory and 78 participants who completed the independent task following their laboratory visit. Further, participants were excluded if they did not meet SST inclusion criteria when undertaking the task in the laboratory (n=14) or independently (n=24; Figure 3).

Regarding the SST, given possible variations in timing related to the operating system and browser being used (particularly during the independent component), the program was designed to capture timing information from the internal timing device, or real-time clock (RTC), of each computer. RTCs are known to be highly accurate [37]. Meta-SSD thus refers to RTC-derived SSD, as opposed to programmed SSD; average SSRT was calculated as mean meta-SSD subtracted from the mean go RT (also timed via the RTC) [21]. There were very strong correlations between laboratory-based meta-SDD (mean 250.23, SD 72.64, range 132.33-484.25 milliseconds) and programmed SSD (mean 249.32, SD 72.65, range 131.25-483.33 milliseconds) (*r*>0.99, *P*<.001) and between independent meta-SSD (mean 223.11, SD 62.86, range 94.45-438.52 milliseconds) and programmed SSD (mean 219.06, SD 62.82, range 92.71-437.50 milliseconds) (*r*>0.99, *P*<.001).

The Kolmogorov-Smirnov test suggested that both independent (*P*=.20) and laboratory-based (*P*=.20) SSRTs were normally distributed. Independent *t* tests were conducted to determine if there were any differences between participants who did or did not meet the SST inclusion criteria. Dependent samples *t* tests were used to consider differences between laboratory-based and independent SST variables. Where multiple *t* tests were employed, a critical *P* value of .005 was adopted to control for multiple comparisons. Effect sizes were computed for *t* tests using Cohen *d* and were interpreted in accordance with Cohen guidelines: small effect=0.20, medium effect=0.50, and large effect=0.80 [38]. Bayesian paired samples *t* testing was additionally conducted to determine the probability of the alternative hypothesis [39]. We adopted the default priors as set by JASP for the Bayesian analyses. In JASP, the prior distribution is defined by a Cauchy distribution centered on zero with a width or scale of 0.707 for *t* tests. Results are presented in terms of Bayes factor BF10, which represents the probability of the observed data given the alternative hypothesis [40]. Bayes factors greater than 1 provide evidence for the alternative hypothesis: values of 1-3 imply anecdotal evidence, values of 3-10 imply moderate evidence, values of 10-30 imply strong evidence, and values of >30 imply very strong evidence [41]. Bayes factors less than 1 provide evidence for the null hypothesis: values of 0.33-1 imply anecdotal evidence, values of 0.10-0.33 imply moderate evidence, values of 0.03-0.10 imply strong evidence, and values of <0.03 imply very strong evidence [41]. Data files are available on the Open Science Framework [42].

### Ethics Approval

This study was performed in line with the principles of the Declaration of Helsinki. The University of Melbourne Human Ethics Committee approved this study (1954220). All participants provided informed consent. In doing so, they acknowledged reading a plain-language statement that explains that aggregated group level data from this study may be published or presented at conferences.

## Results

After exclusions, data from 123 participants (mean age 19.73, SD 1.97, range 18-36 years; 111/123, 90% female) were available for analysis. The Fisher exact test revealed that neither males nor females were excluded to a significantly greater extent in either the laboratory-based (*P*=.66) or independent (*P*=.48) conditions. There were no significant differences between participant samples that did or did not meet the inclusion criteria for the laboratory-based SST with regard to age (*t*_155_=–0.45; *P*=.65; *r*=0.04), years of education (*t*_155_=–0.35; *P*=.73; *r*=0.03), AUDIT score (*t*_155_=0.31; *P*=.76; *r*=0.03), or ASSIST score (*t*_155_=0.10; *P*=.92; *r*=0.01). There were also no significant differences between participant samples that did or did not meet the inclusion criteria for the independent SST on age (*t*_155_=0.73; *P*=.47; *r*=0.06), years of education (*t*_155_=–1.20; *P*=.23; *r*=0.10), AUDIT score (*t*_155_=0.47; *P*=.64; *r*=0.04), or ASSIST score (*t*_24.20_=–1.36; *P*=.19; *r*=0.27). There was no significant correlation between the SSRT—whether derived from the laboratory-based or independent SST—and age, years of education, AUDIT score, or ASSIST score (Table 1).

Average time between SST testing was 3.72 (SD 2.86) days. Table 2 shows the mean (SD) values of laboratory-based and independent SST variables, differences between variables, and the results of Bayesian analyses. Overall, there was a significant positive correlation between laboratory-based and independent SSRT (*r*=0.48; *P*<.001; 95% CI 0.33-0.61). In the group that completed the independent task prior to visiting the laboratory (n=63), there was a slightly stronger association between independent (mean 267.45, SD 35.84 milliseconds) and laboratory-based (mean 239.54, SD 32.37 milliseconds) SSRT (*r*=0.53; *P*<.001; 95% CI 0.39-0.65). There was no significant difference between independent (mean 97.54, SD 1.93) and laboratory-based (mean 97.95, SD 2.01) go accuracy (*t*_62_=–1.57; *P*=.12) in this group. The relationship between laboratory-based and independent SSRT was marginally less strong in the group (n=60) that completed the independent task (mean 257.21, SD 36.59 milliseconds) after visiting the laboratory (mean 241.56, SD 39.03 milliseconds; *r*=0.45; *P*<.001; 95% CI 0.30-0.58). There was a significant difference between independent (mean 96.68, SD 2.62) and laboratory-based (mean 97.96, SD 1.80) go accuracy (*t*_59_=–4.23; *P*<.001) in this group.

**Table 1:** Demographic statistics and Pearson correlation coefficients with laboratory-based and independent stop-signal reaction times (n=123).

Characteristics	Value, mean (SD)	Correlation with SSRT^a^			
		Laboratory-based stop-signal task, *r*	*P* value	Independent stop-signal task, *r*	*P* value
Age (years)	19.73 (1.97)	0.09	.34	0.07	.45
Education (years)	13.50 (1.07)	0.01	.94	0.06	.54
Alcohol Use Disorders Identification Test score	4.60 (4.50)	0.15	.10	0.17	.07
Alcohol, Smoking and Substance Involvement Screen Test score	4.07 (8.68)	0.17	.05	0.16	.08

^a^SSRT: stop-signal reaction time (Go reaction time – meta–stop signal delay).

**Table 2:** Laboratory-based and independent Stop-Signal Task (SST) variables plus differences between variables.

Variables	SST, mean (SD)		*t* test (*df*)	*P* value	95% CI	Cohen *d*	Bayes factor
	Laboratory-based	Independent					
Go accuracy	97.96 (1.90)	97.12 (2.33)	4.12 (122)	<.001	0.43 to 1.24	0.37	242.35^a^
Go reaction time (milliseconds)	490.75 (62.71)	485.56 (60.88)	1.00 (122)	.32	–5.13 to 15.52	0.09	0.16
Go omissions	0.61 (0.99)	0.67 (1.35)	–0.44 (120)	.66	–0.29 to 0.18	0.04	0.11
Go errors	1.43 (1.59)	2.22 (1.97)	–4.24 (120)	<.001	–1.13 to –0.41	0.39	362.27^a^
Go errors reaction time (milliseconds)	431.43 (87.46)	413.44 (82.88)	2.48 (69)	.02	6.64 to 61.05	0.30	2.26^a^
Intraindividual SD	83.03 (26.09)	86.20 (22.60)	–1.27 (122)	.21	–8.10 to 1.76	0.12	0.22
Stop accuracy	50.10 (2.32)	49.61 (2.12)	2.23 (122)	.03	0.05 to 0.93	0.20	1.08
Failed (correct key) stop reaction time (milliseconds)	452.04 (53.27)	446.04 (51.81)	1.26 (120)	.21	–3.22 to 14.56	0.12	0.22
Failed (incorrect key) stop reaction time (milliseconds)	379.58 (58.23)	339.29 (80.89)	2.01 (24)	.06	–1.24 to 99.94	0.40	1.18
Meta stop-signal delay (as timed by each participant’s computer; milliseconds)	250.23 (72.64)	223.11 (62.86)	4.42 (122)	<.001	14.98 to 39.26	0.40	718.50^a^
Stop-signal reaction time^b^ (milliseconds)	240.53 (35.64)	262.45 (36.43)	–6.61 (122)	<.001	–28.48 to –15.36	0.60	9050000^a^

^a^Evidence for the alternative hypothesis.

^b^Stop-signal reaction time = Go reaction time – meta–stop-signal delay.

## Discussion

### Principal Findings

In this study, we sought to ascertain whether performance on a web-based version of the SST differed as a function of the testing environment. Using a counterbalanced design, we investigated if there were differences between variables derived from the task when it was undertaken independently—that is, outside the laboratory, on any computer, and in the absence of researchers—versus when it was performed under laboratory conditions. In keeping with our hypothesis, we found that there was a positive correlation between independent and laboratory-based SSRT. Indeed, this relationship was stronger when the independent SST was completed prior to the laboratory-based measure. Correlations were largely consistent with SSRT test-retest reliabilities reported in other (laboratory-based) studies involving healthy participants (*r*=0.43-0.65) [15,43]. As expected, the independent SST yielded significantly lower go accuracy, increased go errors, and longer SSRTs. Regardless of condition, however, there was no difference in go RT, go omissions, stop accuracy, or intraindividual variability. Bayesian analyses provided very strong evidence in support of the alternative hypothesis in the case of go accuracy, go errors, and SSRT; there was moderate evidence in support of the null hypothesis in the case of go omissions and intraindividual variability. Data could be consistent with either the alternative or null hypothesis in the case of stop accuracy.

Results were largely consistent with an emerging body of evidence examining how the testing environment impacts performance on web-based cognitive tasks. In a study that compared the independent versus fully supervised performance of older adults on web-based tasks assessing attention, memory, and elements of executive function, correlations ranged *r*=0.42-0.64 [32]. In younger participants, correlations of *r*=0.40-0.73 have been reported between test results obtained in person and digitally in assessments of recognition, memory, planning, and attention [31]. As with this study, researchers have noted that these correlations accord with test-retest values reported in the psychometric literature.

Our findings lend some support to the efficacy of employing an independent web-based SST to assess response inhibition in the healthy population. Traditionally, SST data are collected in the laboratory using the same stand-alone computer across all participants so as to reduce variability related to setting and computer hardware or software [23]. This means, however, that participants must visit a research laboratory to take part in studies using this task, and, often, they must be assessed individually. As a consequence, sample size and diversity may be limited. This, in turn, impacts statistical power and the generalizability of the findings. Moreover, these issues may be amplified given the advent of the COVID-19 pandemic and associated government-mandated restrictions. A web-based version of the SST, which ensures that the task is accessible to virtually any person at any location, may minimize these limitations.

Web-based survey–based psychological assessment has been recognized as a cost-effective, efficient, and psychometrically sound means of recruiting large, diverse samples [26,28,44-48]. Web-based versions of cognitive tasks may similarly allow for greater participation in cognitive psychological research. For instance, they will likely enable a greater number of persons located in rural and remote communities to participate in cognitive studies. In terms of SUD-focused research, this may be especially useful in a nation such as Australia, where people living in rural and remote communities consume alcohol at harmful levels or use illicit drugs to a greater extent than those living in urban locations [49]. In fact, the web-based SST was recently used in a study focusing on at-risk drinking and vulnerability for transition to dependence [50]. Researchers secured a large sample (N=814) that was representative of the wider Australian population in terms of country of birth and first language; importantly, more than 10% of the sample heralded from rural or remote regions [50]. Web-based cognitive tasks might additionally facilitate easier access to other hard-to-reach samples—such as older individuals, persons living with mobility issues, culturally diverse groups, or those in treatment [46,47,51]—that tend to be underrepresented in psychological research [52-56].

As web-based studies have been found to foster an increased sense of anonymity and confidentiality among participants, potentially decreasing social response bias and increasing the accuracy of data [47], participants subject to discrimination or stigma might be more willing to take part in cognitive research when protocols are entirely web-based. Where sensitive information pertaining to drug and alcohol use is collected, this is likely to be particularly useful [57]. Finally, web-based cognitive tasks would make collaboration between researchers located in different geographical regions more streamlined, providing participants in any country with ready access to the same protocols.

### Limitations and Further Research

While our results are promising, the independent condition was characterized by a greater number of exclusions (24/157, 15%) than the laboratory-based condition (14/157, 9%). This may have been due to timing and accuracy offsets related to participant concentration or hardware or software variability in uncontrolled testing environments. Nonetheless, exclusion rates were consistent with those cited in other (laboratory-based) SST studies (4%-17%) [17,43,58]. Interestingly, the association between independent and laboratory-based SSRT was stronger when participants completed the task on their own devices prior to visiting the laboratory. This accords with findings in other similar studies examining comparability between independent and laboratory-based cognitive tests [30]. It may be that when undertaking the SST for the first time, participants find laboratory-based testing relatively more stressful—owing to increased researcher supervision, for instance—than when completing the task independently. This may induce a greater degree of task fatigue such that performance is attenuated when it is undertaken the second time. This accords with the significant difference between laboratory-based and independent go accuracy in the group that completed the task in the laboratory prior to undertaking it independently. It would be interesting to examine this proposition further in future.

Regardless, participants exhibited reduced inhibitory control when undertaking the independent SST. This may be owing to the uncontrolled nature of the testing environment in this condition. Response inhibition performance is diminished when attention is compromised, such as when fatigued or under high working memory load [59,60]. To minimize the impact of potential environmental distractors during the independent SST, instructions to participants should include explicit directives to undertake the task at quiet locations. Underperformance owing to nonserious testing attitudes might be an issue [61]. Although this is likely to have been randomized across the whole sample, it would be interesting to determine whether more (or less) impulsive individuals are more susceptible to this phenomenon in future studies. Variability might also emerge owing to the use of different computers and browsers, as well as internet speed [61]. While participants used a surprisingly constrained range of operating systems and browsers when undertaking the task independently (73% used Mac operating systems while Google Chrome or Safari was the browser of choice for 94%), these differed somewhat from those used in the laboratory (Windows, 100%; Google Chrome, 100%). Nonetheless, it is important to remember that behavioral measures of impulsivity are designed to capture transient fluctuations in impulsivity, and variations in performance are expected arise in response to various stimuli and environmental conditions [1,62,63]. As such, SSRT data might be expected to vary between sessions.

Several other limitations should be noted. As the study was advertised via a research participation webpage hosted by the Melbourne School of Psychological Sciences, most participants were undergraduate psychology students. The majority were thus female, and age was positively skewed. Although we can make preliminary assertions regarding the validity of the web-based SST, further research is required to determine whether it satisfactorily reveals changes in response inhibition across the lifespan or if it detects response inhibition deficits in clinical settings. Future studies could consider examining how individuals diagnosed with ADHD or SUDs, for instance, perform on the task as a function of environment or as compared to more traditional forms of the SST. Promisingly, however, the SSRT values reported in this study were consistent with those reported in both a recent meta-analysis [64] and in a psychometric study involving only healthy individuals [65].

### Conclusions

While further testing is required to determine the association between independent and laboratory-based SST variables among individuals diagnosed with clinical conditions, our findings nevertheless suggest that response inhibition can be measured by a web-based SST undertaken outside the laboratory, on any computer, and in the absence of any researcher. The task could, in future, be used as part of a wider battery of assessments conducted entirely digitally and might thus assist in contending with methodological limitations pertaining to sample size and diversity.

## Abbreviations

ADHD: attention-deficit/hyperactivity disorder
ASSIST: Alcohol, Smoking and Substance Involvement Screen Test
AUDIT: Alcohol Use Disorders Identification Test
ITI: intertrial interval
RT: reaction time
RTC: real-time clock
SSD: stop-signal delay
SSRT: stop-signal reaction time
SST: Stop-Signal Task
SUD: substance use disorder

## Data and Materials Availability

The data set analyzed in this study is available in the Open Science Framework repository [42]. The SST can be accessed on the Hester Lab website [35].
